# A Multi‐Pathway Blood‐Based Biomarker Panel Reveals Stereotyped Progression of Blood Biomarker Changes in Alzheimer’s Disease

**DOI:** 10.1002/alz.095559

**Published:** 2025-01-09

**Authors:** Edward N. Wilson

**Affiliations:** ^1^ Stanford University School of Medicine, Stanford, CA USA

## Abstract

**Background:**

Blood‐based biomarkers have emerged as critical tools for detecting Alzheimer’s disease (AD) pathologies decades prior to the onset of clinical symptoms. However, research has been slow to realize the enormous potential of blood‐based biomarkers to capture multiple pathologies and inter‐individual complexities in the biology underlying AD.

**Method:**

We curated a panel of blood‐based biomarkers sensitive to multiple aspects of AD pathology, including vascular, neuronal, immune, along with amyloid and tau. The integrative blood biomarker panel was evaluated in participants along the AD continuum from the Stanford Alzheimer’s Disease Research Center.

**Result:**

Remarkably, participants across the AD continuum exhibit a stereotyped temporal evolution of blood‐based biomarker change such that the first blood‐based biomarkers to change were plasma amyloid, followed by astrocytic GFAP, neuronal p‐tau, neuronal NfL, and finally by vascular marker VCAM1. Deviations to this sequence were rare and indicated other non‐AD neurodegeneration or the presence of familial AD genetic mutations.

**Conclusion:**

The results reveal a natural sequence in pathologic change in AD‐related blood biomarkers. This sensitive blood biomarker panel can be leveraged for precise disease staging and treatment monitoring as we enter a new era of AD therapeutics.

